# Microbial Biotechnology in a Sustainable Future

**DOI:** 10.1111/1751-7915.70492

**Published:** 2026-11-13

**Authors:** Patricia Bernal, Juan Luis Ramos

**Affiliations:** ^1^ Departamento de Microbiología, Facultad de Biología Universidad de Sevilla Sevilla Spain; ^2^ CSIC Granada Spain

## Abstract

This editorial summarizes the main scientific and biotechnological topics that have attracted the greatest interest among our followers, based on the articles they visited most. These topics highlight four interconnected areas: (i) the circular economy, including manufacturing, plastic recycling, and bacterial biopolymer production; (ii) biological engineering and industrial production focusing on synthetic chromosomes, enzyme expression, and antifungal scale‐up; (iii) environmental protection covering climate‐resilient crops, microbial biocontrol, and lindane degradation; and (iv) microbiomes and human health exploring probiotics, myopia treatments, and DNA vaccines. These topics reflect our followers' interest in how biotechnology can address major societal and environmental challenges connected to UN Sustainable Development Goals 2, 3, 9, 12, and 13.

## 1 Introduction: One Field, Connected Challenges

The articles most visited by our followers in the past trimester show that microbial biotechnology impacts environmental protection, sustainable manufacturing, agriculture, and human health. These approaches range from engineering microbial genomes and enzymes to microbial communities and microbiota‐based interventions. Although the studies address different problems, we have found that they share a central idea: biological systems can be understood, redesigned, or managed to provide useful functions; however, there is still a relevant gap between a successful and promising laboratory result and application. Biotechnological processes must consider scale‐up, safety, ecological context, cost, and whether a technology works under real‐world conditions. The most visited studies consider three main aspects: (i) microbes and enzymes can help replace linear, fossil‐dependent production with a more circular use of resources; (ii) biological engineering is becoming more precise, from synthetic chromosomes to tailored protein expression and synthesis of microbial polymers; and (iii) microbiomes matter in both environmental and medical settings, but interventions need careful validation because biological communities' interactions with hosts and the environment are very complex.

In 2017, *Microbial Biotechnology* published a pioneering Special Issue on the alignment of biotechnological processes mediated by microbes with the United Nations Sustainable Development Goals (UN SDGs) (volume 10, issue 5, “The contribution of microbial biotechnology to sustainable development goals”). The most visited articles by our followers during this period were directly related to UN SDG 3 (Good Health and Well‐Being), UN SDG 2 (Zero Hunger), UN SDG 9 (Industry, Innovation and Infrastructure), UN SDG 12 (Responsible Consumption and Production), and UN SDG 13 (Climate Action). The links are potential contributions rather than proof that any technology has already delivered an SDG.

## 2 Building a Circular Bioeconomy

Barcelos et al. (2026), in an editorial introducing the articles published in the Special issue “Biomanufacturing and scale‐up: pathways to sustainable chemicals, biofuels and biomaterials” (https://enviromicro‐journals.onlinelibrary.wiley.com/doi/toc/10.1111/(ISSN)1751‐7915.biomanufacturing‐scale‐up), provide a broad framework for the articles in this specific special issue. The Guest Editors argue that waste carbon streams, including lignocellulosic materials, organic municipal waste, plastics, and simple carbon feedstocks, can be converted into valuable products using enzymes, pure culture microbes, defined consortia, or microbial communities. However, a large share of biologically produced compounds remains at laboratory scale, and the jump from the lab to industrial processes must consider that living cells respond to changing conditions, and that industrial production must integrate feedstock selection, upstream processing, product recovery, modelling, and economic analysis (Akshaya et al. 2026; Crowther et al. 2024; Orfali et al. 2025).

An opinion article independent of the above editorial extends the circular economy argument to plastics (de Eugenio et al. 2026). Enzymes, especially those that degrade PET, may enable biological recycling or upcycling, while biotechnology can also contribute to the production of bioplastics. Yet the authors stress that materials from bio‐based origins or biodegradable do not automatically make them circular. Ways of harvesting, sorting, pretreatment, waste composition, economics, and infrastructure all affect whether the process can be industrially feasible (Olymon et al. 2026). The important connection between the opinion article by de Eugenio and the Editorial by Barcelos et al. (2026) is that the entire production chain must be designed together: feedstock, product, manufacturing, use, and end‐of‐life treatment (Olymon et al. 2026).

Ruiz‐Sáez et al. (2026) presented a specific example of engineering a biological production process, demonstrating that the outer‐membrane protein TolC is essential for mixed‐linkage β‐glucan (MLG) production in *Sinorhizobium meliloti* (Ruiz‐Sáez et al. 2026). Co‐expressing *tolC* with biosynthesis genes and a constitutively active regulator increased MLG production by approximately eightfold compared to the control lacking *tolC*, reaching approximately 10 g/L under bioreactor conditions. This result connects molecular understanding to bioprocess scale‐up, proving that identifying and restoring a missing component of the biosynthetic and export machinery can significantly improve yield and broaden the range of organisms available for industrial production.

Together, these articles show both the opportunities and limitations of circular biomanufacturing. MLG and bioplastics are examples that can contribute to renewable materials, but they are not the only bioproducts to be considered, while waste‐carbon conversion could reduce dependence on fossil feedstocks (Brigham 2018; Soetaert and Vandamme 2011). Nonetheless, as stated in the above‐cited editorial and Opinion articles, environmental benefits must be assessed across the full life cycle, including energy, water, inputs, waste streams, and final disposal. These biotechnological processes align with SDG 12 (Responsible Consumption and Production), SDG 9 (Industry, Innovation and Infrastructure), and SDG 13 (Climate Action).

## 3 Engineering Biological Systems: From Enzymes to Chromosomes

Schramm et al. (2026), in their article “N‐Glycosylation influences the heterologous expression of an unspecific peroxygenase from *Marasmius rotula* in *Saccharomyces cerevisiae* ”, show how genetic engineering of useful enzymes can lead to relevant production changes. In fact, removing glycosylation sites in an unspecific yeast peroxygenase reduced the production of protein, whereas a variant designed to improve glycosylation produced twice as much active enzyme in concentrated culture supernatants as the native version. These findings reinforce Barcelo's editorial regarding the scale‐up message: strain design and process design are interdependent, although protein quantity alone is not sufficient if the product is not active. In this line, de Ovalle et al. (2026) addressed the production of a fungal antifungal protein (PeAfpA) in *Penicillium chrysogenum*, a host selected as a safer production platform than the pathogenic organism that naturally produces the protein. Optimization in small reactors and scale‐up from 0.5 to 5 L identified pH and agitation as important parameters, with dissolved oxygen becoming critical during scale‐up. Yields in bioreactors were tenfold higher than those in flask cultures. This study connects protein engineering and manufacturing directly to agriculture and food safety: reliable production of antifungal proteins may support alternatives to conventional fungicides, although evaluation under field conditions is a must to validate field performance, cost, and regulatory use. This, in turn, is connected with SDG 2 (Zero Hunger).

Lusk and Ehrenreich (2026) went beyond manipulating single genes or controlling processes, either through genetic switches or by controlling physicochemical parameters. In fact, they dig deeper and explain a revolutionary form of engineering: designing and building chromosomes in budding yeast to test how genome sequence, gene content, and chromosome architecture shape cellular traits. Unlike experiments that modify one or a few genes, synthetic chromosomes make large‐scale genome organization an experimental variable (Zhang et al. 2025; James et al. 2025). The authors identified synthetic chromosomes as a platform for understanding eukaryotic biology while noting that predictive design still needs to improve. This work is not itself a near‐term sustainability solution, but it could expand the basic knowledge and tools used in future biomanufacturing.

The set of articles our followers visited in relation to systems engineering forms a continuum from fundamental biology to applied production. Synthetic chromosome research asks how biological systems are organized; glycosylation research shows how protein processing affects expression; and the PeAfpA and MLG studies show that a biological function must be produced reliably at useful scale. This continuum supports SDG 9 (Industry, Innovation and Infrastructure) through scientific and industrial innovation where the resulting enzymes, polymers, or antifungal proteins replace more resource‐intensive processes.

## 4 Agriculture, Climate Resilience, and Environmental Cleanup

We are living in a challenging world, with immediate impacts of global warming (Crowther et al. 2024). Holmes and Matilla (2026) place plant‐associated microbiomes within the challenge of maintaining food production while temperatures and precipitation patterns change. Climate change can alter pathogen distribution, affect plant immunity, and disrupt beneficial microbial communities (Kumar and Mukhopadhyay 2025). These authors discuss strategies including designed synthetic microbial communities, bacteriophages, mycoviruses, microbial biocontrol agents, microbiome engineering, and extracellular‐vesicle‐mediated RNA delivery. They emphasize the difficulty of translating laboratory discoveries into reliable field applications. The central connection to the development of antifungal proteins is the search for biological ways to manage crop pathogens and reduce dependence on chemical pesticides, while both articles make clear that efficacy and practical deployment matter.

Plant roots are not only relevant for plant nutrition but also for distributing microbes in the soil as they can move associated with root development, with colonization of root tips. González De Figueras et al. (2026) explored GMO plants as vehicles to remove pollutants from soil. The authors introduced the bacterial *linA* gene from *Sphingobium japonicum* into *Arabidopsis thaliana* and showed that engineered plants exhibited increased tolerance to lindane and removed more than 90% of the pollutant from the medium within 4 weeks. This work illustrates how a bacterial enzyme can be combined with plant resilience and root interactions to develop phytoremediation strategies for persistent pollutants. This is a promising proof of concept, but not evidence that all contaminated sites can be treated safely or completely; however, previous evidence already exists in the literature for the degradation of hydrocarbons through rhizoremediation (Allamin et al. 2020; Eze et al. 2022; Ghasemi et al. 2026). Our followers understand that plants are not isolated food production units; their outcomes depend on microbes, soil chemistry, climate, contaminants, and surrounding ecosystems. Microbial biocontrol could protect harvests, while engineered plants could help reduce persistent pesticide contamination. Both areas require ecological assessment and field testing to check for unintended effects and to establish whether benefits persist across sites and seasons. These connections are most directly relevant to SDG 2 (Zero Hunger), SDG 12 (Responsible Consumption and Production), and SDG 13 (Climate Action). Lindane remediation, as the mineralization of toxic organic compounds, also relates to protecting environmental quality and reducing exposure to hazardous chemicals.

## 5 Microbiomes and Human Health

Van Tente et al. (2026) examined ways to improve tools available for understanding beneficial bacteria. They focus on expanding genetic tools for *Lacticaseibacillus* and use this microorganism as a model for protein expression. The article is primarily a research tool and uses fluorescent proteins to observe probiotic behaviour and study adhesion, competition, and interactions with hosts. These studies can help to pose questions regarding microbiota‐based therapies.

One of the most surprising findings reported by Li et al. (2026) is the identification of *L. mucosae* MM‐1 from the conjunctival microbiota, and they reported that the bacterium and its metabolite indole‐3‐lactic acid attenuated lens‐induced myopia in mice. The proposed mechanism involves activation of the aryl hydrocarbon receptor and inhibition of TGF‐β/Smad signalling, with changes associated with scleral integrity. These results offer a possible basis for future microbiome‐informed eye‐health research strategies, although the evidence described is from mice and does not establish efficacy in humans yet.

Vicente‐Pazos et al. (2026) compared two DNA vaccine platforms targeting MrkA, a conserved fimbrial subunit of *K. pneumoniae* (Sanchez‐Garrido et al. 2026; Monaci et al. 2025). In a mouse sepsis model, the pVAX1‐based vaccine produced a stronger antibody response than the other vaccine and conferred 80% protection, while the PLASMIVAX‐based vaccine protected only 30% of the animals. Interestingly, the authors demonstrate that the plasmid backbone can influence immune response and protection, even when the target antigen is the same. As a preclinical study, it supports further vaccine development against multidrug‐resistant bacteria but does not establish human efficacy.

Across these health‐focused articles, the microbiome appears to be a source of biological functions, a community that can be studied with improved tools, and a potential target for treatment. The vaccine study complements this topic by showing a different route to infection prevention: rather than altering microbial communities, it trains the immune response against a pathogen. Together, our followers' attention reflects the need for varied approaches to difficult health problems, alongside careful evidence standards. Animal studies, small case series, and research‐tool demonstrations are valuable stages, but they should not be confused with confirmed clinical benefits. The strongest SDG connection of these articles is to SDG 3 (Good Health and Well‐Being), including infection prevention and the responsible development of new therapies.

## 6 Conclusion: Promise Depends on Translation

Taken together, the above articles published in the third trimester of 2026 in *Microbial Biotechnology* show a set of connected strategies rather than a single solution. Biological catalysts can transform feedstocks and materials; genetic engineering can improve production; plant‐microbe research can help protect crops and remediate contamination; and microbiome science can reveal new approaches to health. Several studies report encouraging quantitative results, including increased MLG production, lindane removal, and protective responses in a mouse vaccine model.

The shared lesson is that biotech innovation requires a bridge between biological potential and real‐world performance. Industrial processes need scale‐up and economic and life‐cycle evaluation. Environmental interventions need ecological and field validation. Medical interventions need larger, carefully designed studies and appropriate safety assessment. These requirements do not diminish the promise of the research; they clarify what is needed to turn it into a clear public benefit.

## Author Contributions

**Patricia Bernal:** writing – review and editing, conceptualization. **Juan Luis Ramos:** writing – original draft, writing – review and editing, conceptualization.

## Funding

The authors have nothing to report.

## Conflicts of Interest

The authors declare no conflicts of interest.

## Data Availability

Data sharing not applicable to this article as no datasets were generated or analysed during the current study.
